# Targeted Livestock Metabolomics: A Review of Liquid chromatography–Mass Spectrometry-Based Approaches

**DOI:** 10.1021/acs.jafc.5c16870

**Published:** 2026-06-02

**Authors:** Kangkang Xu, Franz Berthiller, Rudolf Krska, Heidi E. Schwartz-Zimmermann

**Affiliations:** † Institute of Bioanalytics and Agro-Metabolomics, Department of Agricultural Sciences, 27270 BOKU University, Konrad-Lorenz-Straße 20, 3430 Tulln, Austria; ‡ Christian Doppler Laboratory for Innovative Gut Health Concepts of Livestock, 1210 Vienna, Austria; § Austrian Competence Centre for Feed and Food Quality, Safety and Innovation (FFoQSI), 3430 Tulln, Austria; ∥ Institute for Global Food Security, School of Biological Sciences, Queens University Belfast, University Road, Belfast, BT7 1NN Northern Ireland, U.K.

**Keywords:** LC-MS, derivatization, dilute and shoot, validation, analytical methods, animal metabolomics

## Abstract

With the advancement of sophisticated analytical techniques, such as nuclear magnetic resonance spectroscopy and mass spectrometry (MS), metabolomics has become a powerful tool for analyzing and quantifying small molecules in cells, tissues, and biofluids. Among MS-based methods, liquid chromatography–mass spectrometry (LC–MS) is widely used due to its high analyte coverage, sensitivity, and selectivity. Targeted metabolomics quantifies predefined metabolites, in contrast to untargeted approaches that profile all detectable metabolites. This review provides an overview of targeted LC–MS methods and applications in livestock metabolomics, focusing on ruminants and swine, and covering research from the past decade. We discuss sample preparation, LC–MS instrumentation, and method validation, as well as emerging trends including combined LC techniques, integration of targeted and untargeted approaches, and multiomics studies. Current limitations, future directions, and a general workflow for method development are also addressed.

## Graphical abstract

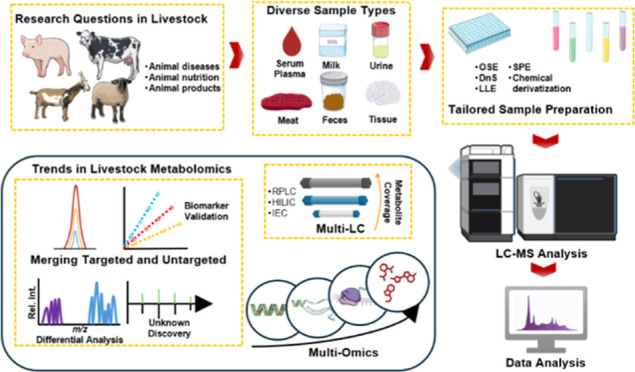

## 1 Introduction

Metabolomics is the comprehensive study of small-molecule metabolites within cells, tissues, or organisms.^1–3^ A metabolome typically consists of several thousand low-molecular-weight metabolites with diverse physicochemical properties, differing in size, molecular weight, volatility, hydrophobicity and solubility. Their concentration ranges span 6 orders of magnitude.^4^ Metabolomics captures a dynamic profile of metabolic products that reflect ongoing physiological and biochemical processes. By analyzing these metabolites, metabolomics provides insight into the functional state of a biological system and its response to genetic, environmental, or pathological influences. It represents the end point of the “omics” cascade, offering a chemical reflection of a molecular phenotype and therefore bridging the gap between genotype and phenotype.^5^ Metabolomics serves as a valuable complement to other omics techniques, such as genomics, transcriptomics, and proteomics, with a growing emphasis on integrating the multiomics data sets.^6–8^ Beyond traditional core “omics,” emerging omics technologies and associated platforms are rapidly expanding. These include lipidomics, which focuses on the identification and quantification of diverse lipid species; microbiomics, which investigates an organism’s microbiota and microbiome; and epigenomics, which examines genome-wide DNA modifications that regulate gene activity.^9,10^

Metabolomics has found broad applications in various fields, such as environmental toxicology,^11^ biomedical research for disease monitoring and prediction,^12,13^ food and nutritional analysis for discovering and validating dietary biomarkers,^14^ and agricultural and plant science for assisting breeding of resistant varieties or developing new bioproducts.^15^ Metabolomics studies are separated into two distinct categories: targeted and untargeted metabolomics. Untargeted metabolomics is a discovery-driven approach which is carried out without predefined targets or assumptions. It aims to detect and analyze all measurable metabolites in a biological sample.^16^ In contrast, targeted metabolomics is a hypothesis-driven approach and allows researchers to identify and quantify predefined sets of known metabolites.^17^ Each approach has intrinsic advantages and disadvantages, and they are often used in an integrated manner.^18^

The advent of universal analytical techniques such as nuclear magnetic resonance (NMR) spectroscopy and mass spectrometry (MS) enabled the detection of a wide range of metabolites. Compared to NMR, MS can profile a large number of metabolites across a wide concentration range, with low-resolution platforms like triple quadrupole mass spectrometers (QqQ) offering high sensitivity, cost-effectiveness, and robustness. While complementary, the limited availability of two expensive techniques in one lab typically restricts the application to either NMR or MS. Advantages of NMR are high reproducibility, little to no sample preparation, speed of analysis and lower costs per sample. In contrast, MS techniques score with high sensitivity and far greater metabolite coverage. Thorough comparisons of the techniques are presented in comprehensive reviews.^19,20^

In targeted metabolomics, liquid chromatographic (LC) separation followed by tandem mass spectrometric detection (LC–MS/MS) in selected reaction monitoring (SRM) mode is the gold standard for quantifying hundreds of metabolites due to its high sensitivity, wide dynamic range, and good reproducibility.^21^ Complementary to that, flow injection analysis-tandem MS (FIA-MS/MS) enables faster analysis without chromatographic separation while maintaining low detection limits.^22–25^

Sample preparation and chromatographic separation are two critical steps in LC–MS based analysis, as they can reduce mass spectrometric matrix effects (which manifest themselves as signal enhancement or suppression in the presence of sample matrix compared to pure solvent^26^), improve ionization efficiency, and enhance the detection of low-abundance metabolites. Reversed-phase liquid chromatography (RPLC) is widely used in metabolomics and is particularly suited for lipidomics due to its hydrophobicity-based separation mechanism, ensuring efficient resolution of apolar lipids. Hydrophilic interaction liquid chromatography (HILIC) serves as a valuable complementary technique for analyzing polar metabolites without the need for chemical derivatization. Additionally, ion-exchange chromatography (IEC) can be used to separate highly polar and ionizable molecules. Owing to the wide range of physicochemical properties, structural diversity, and broad concentration range of metabolites, no single LC–MS method can comprehensively cover the entire metabolome. Consequently, the combination of two or more distinct orthogonal LC methods in large-scale targeted LC–MS approaches improves metabolite coverage compared to traditional targeted methods.^27,28^

The review by Goldansaz et al.^20^ provides a quantitative overview of livestock metabolomics prior to 2015, highlighting the widespread use of LC–MS and underlining the need for greater emphasis on absolute metabolite quantification. Since then, targeted LC–MS has become increasingly popular in livestock metabolomics. A growing number of studies in livestock science demonstrate that targeted metabolomics and metabolite-based phenotyping (metabotyping) can provide practical benefits across farming, veterinary care, and livestock science. These studies showcase the versatility of metabolomics for predicting and diagnosing animal diseases,^29,30^ evaluating effects of dietary feed additives,^31,32^ fertility,^33,34^ and nutritional quality of milk,^35^ characterizing carcass quality,^36,37^ assessing feed regimes on meat production,^38^ and for exploring markers of methane emissions in ruminants.^39,40^ Absolute quantification can be achieved in targeted metabolomics, enabling comparisons of metabolite concentrations across subjects, platforms, laboratories, and countries, supporting the establishment of normal and abnormal metabolite ranges for disease diagnosis, prediction, and other relevant production metrics.^20^ Furthermore, acquiring quantitative physiological concentrations facilitates biomarker discovery.^41,42^ Advances in mass spectrometry, liquid chromatography, and data processing have significantly enhanced the sensitivity, throughput, and reproducibility of targeted metabolomics.^43^ These improvements made the approach more accessible to a wider range of applications in livestock science. Consequently, targeted metabolomics has expanded beyond specialized laboratories and is now commonly used for routine quantitative analysis. Commercial kits also enable quantitative analysis of hundreds of metabolites in a variety of livestock matrices, although they were originally designed for human samples. Additionally, several high-quality, large-scale studies have assessed different LC techniques and fine-tuned MS parameters, providing essential guidance for researchers to establish custom quantitative targeted LC–MS/MS methods.^44–46^ Large-scale targeted LC–MS/MS methods in other fields, such as in human and rodent research,^47–55^ have contributed rich data sets on LC and MS/MS settings, supporting method development across the analytical and livestock communities.

Despite the adoption of well-established targeted LC–MS methodologies from other research fields, livestock metabolomics presents unique analytical challenges. Compared to human studies, livestock research involves a broader diversity of biological matrices, adding meat, rumen fluid, eggs, or digesta to the commonly used matrices of blood/plasma, urine, and feces. These animal matrices are unique, and also exhibit distinct metabolite patterns. Depending on the livestock species and chosen matrix, also sampling might be far more complex than for human metabolomic studies.

While the review by Goldansaz et al. comprehensively examined diverse analytical platforms,^20^ the present work serves as a follow-up and narrows the focus to targeted LC-MS-based livestock metabolomics, critically assessing current methodologies, trends, and applications. To this end, we collected the relevant literature on the topic that had been published over the past ten years. On the one hand, the strengths and limitations of commercial kits are highlighted. On the other hand, available targeted LC–MS methods are systematically compared in terms of sample preparation, LC–MS parameters, and method validation. This review summarizes the livestock categories and sample types examined in the literature, and explores emerging trends such as multiomics and integration of targeted and untargeted metabolomics approaches, while highlighting key methodological gaps and challenges. Finally, practical recommendations are provided to guide researchers in selecting and applying targeted LC–MS approaches for livestock studies.

## 2 Article Search, Selection and Classification

Web of Science (https://www.webofscience.com/) was chosen as the main platform for literature search, as the initial comparison of search results across various search engines (e.g., Web of Science, PubMed and Google Scholar) showed similar outcomes. The following keywords were used.

1. liquid chromatography mass spectrometry AND targeted metabolomics
2. liquid chromatography mass spectrometry AND untargeted metabolomics or nontargeted metabolomics
3. cow* or cattle or bovine or bovid*
4. horse* or equine or equid*
5. sheep* or ovine or small ruminant*
6. goat* or caprine
7. pig* or piglet* or porcine or swine

The search was conducted using 1 or 2 combined with 3, 4, 5, or 6, respectively. The asterisk wildcard character enables searching for all possible suffix variations, while “OR” is used inclusively to retrieve results containing one or more alternative terms. The literature search took place on February 2, 2025. Initially, we inspected the titles and abstracts, checked the availability of the full text, and excluded reviews from the list of articles. Additionally, references from pivotal studies or reviews were also included for further evaluation. Each article was screened based on pre-established criteria, while the incompatible articles were eliminated from the article database. Figure 1 summarizes the article search and screening strategy.

**Figure 1:**
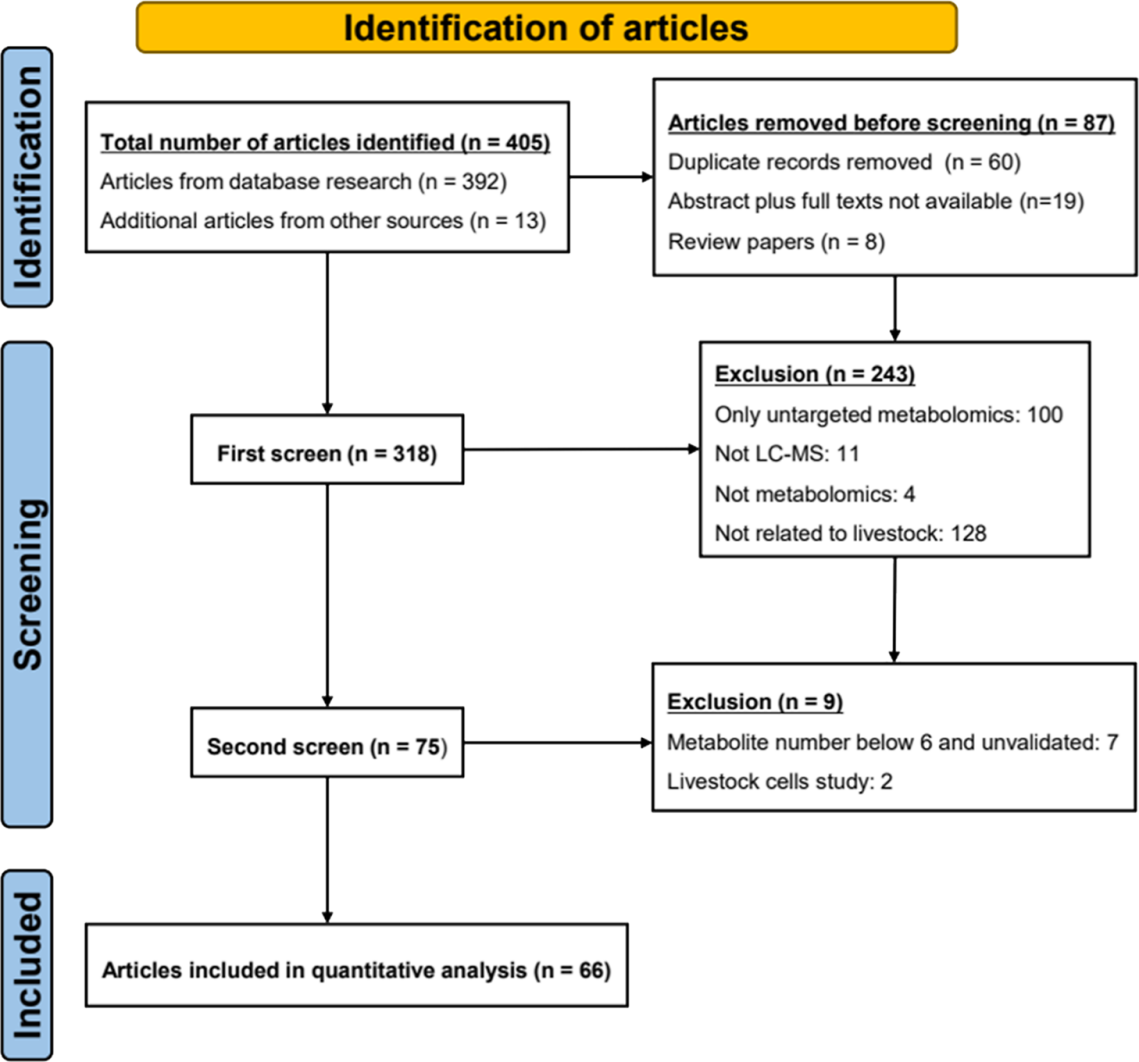
PRISMA diagram illustrating the search and screening strategy for articles on targeted metabolomics by liquid chromatography mass spectrometry.

Both search strategies for targeted and untargeted LC–MS yielded a comparable number of identified publications, with targeted and untargeted accounting for 49% and 51% of total articles, respectively. To be included in the set of targeted metabolomics articles selected for detailed evaluation, studies had to be original, peer-reviewed research articles published in English language. Scientific articles that were not relevant to livestock metabolomics, used only untargeted metabolomics approaches, or did not employ LC–MS were excluded from the literature database. We further narrowed down the number of articles by excluding those involving targeted LC–MS methods with a metabolite number <6, or studies focusing on animal cells. A total of 66 articles were examined for information on animals, sample types, targeted metabolite classes, metabolite number, sample preparation, LC–MS methods, and research aims. Those 66 articles were also used to identify trends in research questions, and to evaluate the frequency of combined use of targeted and untargeted metabolomics, and of multiomics research. Regarding research aims, we categorized the articles into animal health, animal nutrition, animal production, animal reproduction, human health (animals as a model to investigate human health), animal products, animal physiology, and methodology. We followed the same seven research aims as defined by Goldansaz et al.,^20^ with an additional category for methodology, which includes articles primarily focusing on the development and validation of LC–MS methods for targeted livestock metabolomics. Twenty-five articles used commercial kits for targeted metabolomics, which were not included for further systematic comparison. Articles using identical sample preparation and LC–MS methods were combined to one representative methodology cluster. In total, the 66 articles yielded 36 methodology clusters as indicated in Table S1. One methodology cluster can contain multiple sample preparation and/or LC–MS methods. Subsequently, all individual 51 LC–MS methods employed in the 36 methodology clusters were systematically compared in terms of sample preparation, chromatographic separation, MS instrumentation, and validation (Table S2).

## 3 Overview of Current Analytical Methods

### 3.1 Commercial Metabolomics Kits

Commercial metabolomics kits, such as offered by Biocrates (Innsbruck, Austria), TMIC (Edmonton, Canada) or SCIEX (Marlborough, MA, US), were originally designed for human serum and plasma analysis. Considering the comparable concentration ranges and metabolite compositions of human and animal plasma,^28^ commercial metabolomics kits are also widely used for the quantitative analysis of metabolites in different biological matrices of livestock. Of the 25 selected articles using commercial kits, 24 employed a Biocrates kit, whereas only one article used the TMIC Prime kit. Among these, the Absolute IDQ p180 kit was the most frequently employed assay in targeted animal metabolomics (63%), followed by the MxP Quant 500 kit (25%), Absolute IDQ Bile Acids kit (8%), and Absolute IDQ p400 HR kit (4%). The Absolute IDQ p180 kit covers 186 metabolites from 7 compound classes, including amino acids, biogenic amines, acylcarnitines, glycerophospholipids, sphingolipids, and hexoses. The MxP Quant 500 kit is used to identify and (semi)­quantify 630 metabolites from 26 classes. Sample preparation is conducted in 96-well plates. For both kits, derivatization using phenyl isothiocyanate (PITC) is performed for the subsequent detection of amino acids, amino acid related compounds and biogenic amines, that would otherwise be poorly retained on an RP column. To correct for the variations introduced during sample preparation and to account for mass spectrometric matrix effects, the 96-well plates are delivered with a set of isotopically labeled internal standards (ISs) present in each well that undergo all steps of sample preparation.

Biocrates kits employ FIA-MS/MS and LC–MS/MS to maximize metabolite coverage and analytical throughput. FIA-MS/MS is typically used for lipid classes, including acylcarnitines, phosphatidylcholines, lysophosphatidylcholines, sphingomyelins, and ceramides, as well as for hexoses. In contrast, LC–MS/MS is applied to compound classes such us amino acids and biogenic amines to improve chromatographic separation, to enhance ionization and by that achieve lower limits of detection, to reduce mass spectrometric matrix effects, and to ensure accurate quantification of isomeric compounds. For compounds measured by RPLC-MS/MS, quantification is based on solvent calibration curves of (equally derivatized) authentic reference compounds relative to, if available, dedicated isotopically labeled ISs or, if not available, relative to structurally similar labeled ISs. In the case of metabolites measured by FIA-MS/MS, metabolite concentrations are estimated by comparing the signal intensity of each metabolite to its allocated IS.

As well-established, readily accessible targeted assays, commercial kits save researchers time in method development and validation. These kits are surprisingly versatile and applicable across a variety of sample types and species. They have been used in diverse targeted livestock metabolomics studies. Examples include biomarker discovery in disease diagnosis,^56,57^ prediction of subclinical disease,^29,58^ exploration of dietary effects on the tissue/body fluid metabolome,^31,59^ and metabolic profiling in biological fluids during physiological perturbations.^60,61^ Kits were also integrated into multianalytical platform studies to expand the metabolic coverage and identify more discriminative metabolites between control and treatment group.^39,40^ Moreover, kits can serve as reference for internal consistency assessments to evaluate values obtained through other analytical methods, especially in cases where certified reference materials (CRMs) are unavailable. For instance, Leuthold et al.^62^ used a Biocrates kit to cross-validate an untargeted metabolomics method for porcine kidney tissue, yielding good correlations. With recent technological advancements, the latest Biocrates kit–MxP Quant 1000–enables the identification and (semi)­quantification of up to 1233 metabolites from 49 metabolite classes.

However, commercial kits also have certain drawbacks. First, they are expensive. Second, derivatized analytes are unstable and derivatized plates cannot be frozen for storage, which requires immediate analysis or repeated sample preparation using a new kit. Third, the derivatization step—including multiple pipetting and evaporation actions—adds complexity to the sample preparation. Additionally, in FIA-MS/MS-based lipid quantification, concentrations are estimated through a one-point calibration curve established from ISs, where only a few ISs are available for a whole metabolite class. This consequently impairs accuracy, especially for compounds experiencing matrix effects, showing nonlinear responses, and/or occurring in a concentration range not covered by the ISs.^63,64^ Validation data have been published only for human milk so far.^65^

Apart from Biocrates kits, other commercial targeted metabolomics platforms are available, including TMIC targeted kits, SCIEX Lipidyzer for lipidomics,^66^ and various class-specific kits (e.g., amino acid, bile acid, or acylcarnitine panels) offered by different manufacturers. These platforms often focus on specific metabolite classes or analytical workflows, in contrast to the more comprehensive kits offered by Biocrates. When selecting a commercial metabolomics kit for targeted livestock metabolomics, several factors should be considered. These include metabolite coverage relative to the research question, instrument capability,^67^ suitability for specific livestock matrices (e.g., feces, serum, rumen fluid), IS availability, quantitative capability (absolute or semiquantitative), validation status, analytical throughput, and cost consideration. While comprehensive kits provide broad multiclass coverage, smaller kits tailored to specific metabolite classes may offer advantages in sensitivity or cost efficiency.

### 3.2 Sample Preparation Strategies

#### 3.2.1 Metabolite Extraction

A well-designed sample preparation process helps to remove unwanted compounds, minimize matrix effects, and/or convert metabolites into a form compatible with the intended analytical techniques in complex biological samples.^68,69^ Selecting an optimal extraction method depends on various factors, such as macromolecule content, metabolite polarity, and analyte concentrations in biological samples. Uneven sample matrix complexity across animal samples such as blood, plasma, urine, feces, milk, rumen fluid, eggs, and saliva introduces significant technical bias in targeted LC–MS metabolomics. This bias arises partly from selective metabolite losses during sample preparation, which can disproportionately affect certain compound classes. As a result, the risk of systematic under- or overestimation of metabolite concentrations increases, especially when uniform extraction strategies are applied across different matrices.^70^

In this chapter, we compare the sample preparation strategies employed in the selected 51 methods. One the one hand, sample preparation for metabolomics can be a simple dilution or relative enrichment of the metabolites of interest by removal of matrix, such as tissue debris or precipitated proteins, by filtration or centrifugation. On the other hand, it can also involve metabolite extraction methods like organic solvent extraction (OSE), liquid–liquid extraction (LLE), as well as solid phase extraction (SPE).^71^ Dilute-and-shoot’ (DnS) is defined as the dilution of a sample matrix with an appropriate solvent prior to analysis. However, the definition of DnS has evolved over the years and tends to be inconsistent in the literature, as several publications claiming use of the DnS approach actually include additional steps such as solid–liquid extraction or deproteinization as outlined by Greer et al.^72^ In this review, we stick to the original definition of DnS and reserve it for processes in which solvent was added to the liquid sample without removal of compounds before analysis. Sample preparation consisting of deproteinization by precipitation of proteins with organic solvent and dilution is referred to as “protein precipitation and dilution”, whereas OSE based sample preparation involves additional steps such as solvent evaporation and reconstitution.

Due to its versatility and ease of use, OSE based sample preparation is the most widely adopted method for sample treatment across all biospecimens.^71^ This aligns with our findings, where 60% of the targeted LC–MS based methods used an OSE based protocol and chemically diverse metabolites were extracted ranging from polar amino acids and carboxylic acids to nonpolar compounds like bile acids and long-chain fatty acids. The utilized solvents were methanol, acetonitrile (ACN), isopropanol (IPA), or their mixtures with water. OSE based sample preparation was broadly applied to diverse sample types, including body fluids and tissues. However, organic solvents play a different functional role based on the matrix. In body fluids, they primarily facilitate macromolecule precipitation (e.g., proteins and DNA) and enzyme inactivation. In contrast, in tissue samples, they primarily aid in metabolite extraction and dissolution, as well as in the release of protein-bound metabolites and enzyme inactivation.

In addition to DnS that, in the original strict sense, includes only sample dilution,^72^ the protein precipitation and dilution strategy involving centrifugation to remove solid debris and dilution to adjust the concentration of the targeted metabolites has gained popularity in multianalyte LC–MS analysis.^73,74^ Protein precipitation and dilution was employed by 10% of the total methods prior to targeted LC–MS analysis. It was mainly applied to biological fluids, including serum, plasma, and rumen fluid (see Tables S1 and S2). Simple DnS was applied in one article for analysis of metabolites related to nitrogen status in urine.^75^

Although chemically diverse and containing numerous metabolites contributing to background signals, these matrices are often amendable to simple sample preparation steps such as protein precipitation and dilution (serum, plasma, rumen fluid) or DnS (urine). On the contrary, more complex samples such as e.g. tissues typically require additional sample cleanup strategies to reduce mass spectrometric matrix effects.^72^ Advantages of the DnS and protein precipitation and dilution approaches over other protocols are their simplicity, low analyte loss, efficient sample processing, and broad coverage of metabolite classes. Integration of DnS and protein precipitation and dilution with LC–MS/MS, particularly on triple quadrupole (QqQ) instruments, has given rise to the development of multiclass, multianalyte methods across various matrices and research fields.^72^ A typical DnS workflow using ACN and centrifugation was applied to bovine urine samples prior to the HILIC-MS/MS analysis of urinary purine derivatives.^75^ In another recent study conducted by Xu et al.,^28,76^ a simple approach including precipitation of plasma proteins with IPA/water (80/20, v/v), centrifugation and dilution, was employed to quantify 235 porcine plasma metabolites across 19 chemical classes. Both methods showed very good repeatability, accuracy, and recovery for most analytes, which can be partially attributed to the robust and straightforward protocol. However, both DnS and protein precipitation plus dilution face the challenge of matrix effects resulting from highly abundant coextracted compounds, which may hinder the detection of low-concentration compounds in biological matrices,^28^ so that a cleanup step might be required for determination of certain low-abundance metabolites.

LLE and SPE accounted for 13% and 4% of the total methods, respectively, while 2% of the methods did not specify the type of sample preparation. Seven methods used LLE to separate compounds based on polarity differences between two immiscible solvents. Among them, 71% utilized either a MeOH/chloroform/water or ACN/chloroform/water system modified from a classic LLE method.^28,77^ After phase separation, lipids are enriched in the organic (chloroform) layer, while the polar layer contains predominantly more hydrophilic metabolites. Additionally, LLE is a versatile extraction approach and can be tailored to a specific metabolomics application by selectively collecting the phase, either aqueous or organic, that contains the metabolites of interest. It was used to extract polar metabolites, including taste-active compounds, such as amino acids and nucleotides, from bovine meat tissue.^36^ Likewise, derivatized carboxylic acid-containing metabolites associated with glycolysis, ketogenesis, and the Krebs cycle were extracted from swine sperm lysate using ethyl acetate as organic solvent for LLE.^34^ In contrast, LLE was also employed to extract apolar sphingolipids, phospholipids, and cholesterol from multiple bovine tissues and digesta (e.g., liver, muscle, fat, duodenum content).^78–80^ SPE was exclusively used for purification and concentration of metabolites from body fluids within our survey, including plasma and serum. In one SPE-based workflow, an initial organic solvent extraction step was required to both extract the metabolites and precipitate proteins prior to SPE cleanup.^81^

Finally, methodology clusters that use two or more methods might also have dedicated sample preparation methods for specific submetabolomes or sample types of interest. In one such cluster,^82^ ice-cold ACN was used to extract polar metabolites from bovine plasma, followed by centrifugation and up-concentration. Meanwhile, the plasma lipidome was extracted by a modified methyl *tert*-butyl ether (MTBE)-based liquid–liquid extraction,^83^ where plasma was mixed with methanol and MTBE, followed by water-induced phase separation. In another example, a single-step methanol precipitation was used to precipitate proteins and extract the analytes from plasma, while a single-step protein precipitation and extraction using acidified ACN was employed for extracting sulfur-containing metabolites.^84^ Apart from clusters with dual sample preparation protocols, one study^33^ adopted a sample preparation protocol from a previous study,^85^ in which 4 common protocols including protein precipitation, RP-SPE, high-pH RP-SPE, and phospholipid-depletion solid-phase extraction (PD-SPE) using a Sigma-Aldrich HybridSPE-Phospholipid 96-well plate were compared for the LC–MS/MS quantification of bile acids in blood. As a result, PD-SPE using ACN/water/formic acid (70.0/29.8/0.2, v/v/v) as both conditioning and washing solvent effectively removed phospholipids and outperformed the conventional protein precipitation and SPE method in terms of analyte recovery and reproducibility.

In conclusion, these examples illustrate that sample preparation in targeted metabolomics can be tailored to extract specific metabolite classes or adapted to different sample types. However, implementing multiple preparation steps complicates the workflow, increases the risk of errors, and reduces overall throughput – particularly in routine analyses involving large numbers of samples.^86^ Together, these findings highlight the importance of comparative evaluation of sample preparation strategies, as selecting the most appropriate protocol is critical for optimizing metabolite coverage, analytical sensitivity, and reproducibility in targeted LC–MS workflows.

#### 3.2.2 Chemical Derivatization

Eight of the 51 methods covered in this review employed derivatization during sample preparation. Chemical derivatization increases the hydrophobicity of polar or ionic compounds, which are otherwise poorly retained in RPLC. Derivatization provides several advantages, including expanded metabolic coverage, increased detection sensitivity, better separation and enhanced metabolite identification due to specific mass shifts.^87^ For these reasons, derivatization is an indispensable step in commercial kits. Apart from PITC that is widely used for derivatization of primary amines (see Section 3.1), several other reagents were used to target specific functional groups of metabolites (Table S2). Three out of the eight methods employing derivatization used 3-nitrophenylhydrazine (3-NPH), a reagent capable of derivatizing carboxylic acids, aldehydes, and ketones prior to LC–MS/MS analysis.^88^ In that respect, Schwartz-Zimmermann et al.^89^ compared the performance of two RPLC-MS/MS methods using aniline or 3-NPH as derivatization reagents for the quantification of carboxylic acids in bovine feces and ruminal fluid, with anion exchange chromatography coupled to high resolution mass spectrometry (AEX-HRMS) as the reference method. The study demonstrated that derivatization with 3-NPH provided superior performance compared to aniline in the quantitative metabolic profiling of carboxylic acids in animal matrices. To broaden the submetabolome coverage, multiple reagents can be used in a single method. Trudeau et al.^90^ used dansyl chloride to derivatize amino acids and 2-hydrazinoquinoline to derivatize carboxylic acids, aldehydes, and ketones, respectively.

However, chemical derivatization also comes with disadvantages, especially the inconvenience of the derivatization procedure itself. Compared to conventional label-free methods, chemical derivatization extends and complicates sample preparation, thereby reducing overall throughput and impairing repeatability. Additionally, the stability of some derivatized metabolites is short-lived, necessitating immediate analysis or repeated sample workup.^91,92^ Derivatization agents might also introduce impurities interfering with LC–MS analysis. Regardless of these limitations, several strategies can enhance the robustness and reliability of derivatization-based workflows. Employing ISs that undergo identical derivatization reactions helps compensate for variability introduced during sample preparation and contributes to the compensation of matrix effects. In addition, sample preparation automation and postcolumn online derivatization can reduce variability,^93^ even if the latter does not improve separation. Testing the stability of derivatized analytes, careful validation of derivatization efficiency, and the inclusion of quality control samples improves method reproducibility. Above all, the widespread use of chemical derivatization in targeted LC–MS methods, including kits, underscores its advantages in livestock metabolomics, which often outweigh its limitations.

### 3.3 From Single to Multi-LC Separation

Selectivity in LC is determined by the stationary phase material and can be further fine-tuned by adjusting the eluent composition, gradient conditions, and pH value. Given the wide range of columns available and the complexity of separation mechanisms, selecting a suitable column and optimizing chromatographic conditions remains a significant challenge for researchers, particularly in large-scale metabolomics methods targeting hundreds of chemically diverse metabolites. In light of several large-scale multi-LC comparison studies,^44–46^ we compared the 51 LC methods covered in this review, providing advice on LC method selection. Columns are categorized into four groups: RP, HILIC, IEC, and not given (NG). Within each group, further subdivision depends on the stationary phase material and/or separation mechanism. A summary of the individual columns used is displayed in Figure 2, with several studies using more than one column.

**Figure 2:**
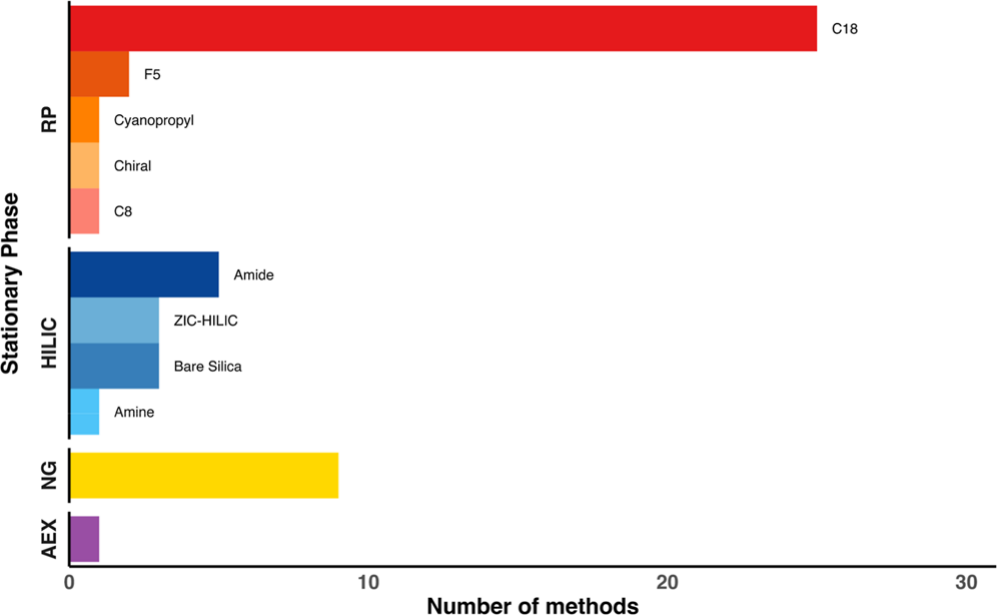
Overview of the usage frequency of each column type in our survey for targeted livestock metabolomics. RP: reverse-phased lipid chromatography; HILIC: hydrophilic interaction lipid chromatography; AEX: anion exchange chromatography; F5: pentafluorophenyl propyl; NG: not given.

#### 3.3.1 Reversed-phase Liquid Chromatography

RPLC was the most widely used technique (58%) among the 51 LC–MS studies included, followed by HILIC (24%) and AEX (2%), while 17% of methods did not specify LC setups. This reflects earlier reports stating that RPLC is considered the most prevalent LC technique employed in metabolomics studies in general.^27,94^ The actual prevalence of RPLC is even higher in livestock studies, given that it is typically used as a standard LC method in commercial kits. 48% of all targeted LC–MS methods employed C18 columns. While C18 columns contain similar octadecyl-bonded ligands, the overall analytical performance can vary across various stationary phase materials. The variation is attributed to modifications involving factors such as the type of silica used, the quantity and nature of residual silanols, end-capping techniques, ligand bonding density, pore size, and other proprietary features.^94^ Apart from the use of RP-C18 columns, two RPLC-MS-based methods utilized pentafluorophenyl (F5)-bonded stationary phases. Compared to alkyl phases, F5-bonded stationary phases provide multiple interaction sites, including π–π interactions and enhanced dipolar interactions. Additionally, weak ion-exchange interactions may arise from residual silanols or intentionally incorporated ionic groups as well.^95^ Consequently, fluorinated phases facilitate the usually challenging separation of polar and basic compounds in RPLC workflows.^46,96^ An example is the application of an RP-F5 method to quantify taste-active metabolites in bovine meat, including polar free amino acids, nucleotides, hypoxanthine, and succinic acid.^36^ Overall, RP columns are widely used to separate nonpolar and medium polar metabolites in livestock science, such as fatty acids,^81^ bile acids,^97^ and oxylipins.^98^ Chemical derivatization extends the number of compounds accessible to RP separation to polar and even ionic compounds.

#### 3.3.2 Hydrophilic Interaction Chromatography

Based on our analysis, HILIC is the second most used LC technique in the field after RPLC, accounting for 24% of the total methods. HILIC is widely used in metabolomics for separating polar analytes without derivatization.^27^ HILIC-MS methods cover a broad range of polar metabolites, for example amino acids, amino acid related compounds, and biogenic amines,^99^ sugars,^100^ and nucleobase derivatives.^75^ One HILIC-MS/MS method using a neutral BEH HILIC column was adapted to quantify choline-containing metabolites, such as lysophosphatidylcholine, phosphatidylcholine, and sphingomyelin in bovine tissues.^28,78^ Phospholipids were separated based on the polar head groups, promoting the retention. The high percentage of ACN under HILIC conditions also enhanced the sensitivity and improved peak shapes for the choline-containing metabolites.^101^ Strikingly, several studies implemented large-scale targeted HILIC-MS methods for the quantification of more than 200 metabolites. For instance, Broadwin et al. adopted a targeted HILIC-MS/MS method for the profiling of 258 polar metabolites in porcine blood and myocardial tissue.^102^ Similarly, Li et al. utilized an amide HILIC column coupled to tandem MS to quantify over 300 metabolites related to 35 metabolic pathways in swine heart tissue.^103^ In addition, a bare silica based HILIC-MS/MS method was used to determine 108 metabolites in plasma from different animal species.^28,76^ Despite its effectiveness in covering polar metabolites, HILIC remains relatively underutilized compared to RPLC for several reasons.^27,104^ Researchers are generally more familiar with RPLC, a more established method with a long-standing history.^105^ Second, RPLC offers a greater variety of column materials compared to HILIC.^106^ In addition, RPLC is often perceived to have a more robust performance than HILIC.^50,107^ This perceived robustness of RPLC is attributed to its relatively simple hydrophobic-based separation mechanism, which provides predictable retention behavior and greater tolerance to variations in mobile phase composition. In contrast, HILIC has a mixed retention mechanism, including hydrophilic partition, adsorption, and electrostatic interactions.^108^ Consequently, chromatographic separation in HILIC is more susceptible to changes in pH, mobile phase/buffer, and water content compared to RPLC. Additionally, HILIC also needs longer equilibration times than RPLC to allow sufficient formation of a stable, water-rich layer on the stationary phase, which acts as the partitioning medium for analytes. Special emphasis should be laid on matching the injection solvent to the mobile phase at the start of the run. This might be problematic as some very polar analytes are hardly soluble in the starting mobile phase of HILIC, containing high proportions of organic solvent. This might be overcome by using smaller injection volumes than needed for RPLC, hence sacrificing sensitivity.^109^ Finally, HILIC typically requires a certain amount of salts in the mobile phases, that might lead to clogging of columns.

#### 3.3.3 Ion Exchange Chromatography

IEC was used by only one research group working on targeted animal metabolomics, e.g.,.^89,110–112^ The separation mechanism of IEC is based on electrostatic interactions between analyte ions (or highly polar metabolites rendered ionic by the mobile phase) and an oppositely charged stationary phase. IEC is divided into cation-exchange chromatography and AEX, with AEX representing the predominant mode applied in metabolomics analyses. Although HILIC and IEC exhibit some overlap in the metabolites they target, the distinct mechanism of IEC enables an expanded metabolome coverage compared to RPLC and HILIC, particularly for highly polar and charged molecules.^113^ In HILIC, peak tailing and broadening can arise from multiple factors, including electrostatic interactions and slow desorption kinetics,^109,114^ and are particularly pronounced for highly polar anionic compounds such as phosphorylated metabolites and poly­(carboxylic acid)­s^44^ that constitute a large proportion of glycolysis and tricarboxylic acid cycle (TCA) intermediates. AEX is perfectly suited for separation of various types of short chain carboxylic acids (<C10) and polar phosphorylated compounds, while providing well-defined peak shapes. This was demonstrated in several studies, where an AEX-HRMS method was routinely used to identify and quantify nucleotides, sugar phosphates, and carboxylic acids in diverse biological fluids of livestock.^32,111,112,115–117^ Moreover, AEX-HRMS was also implemented as a reference method to compare two derivatization protocols for the quantitative determination of various carboxylic acids in animal samples.^89^

#### 3.3.4 Combination of Chromatographic Techniques

A metabolome consists of a vast number of metabolites displaying diverse chemical and physical properties; for instance, the human metabolome is estimated to contain over 270,000 metabolites.^118^ These metabolites span multiple compound classes, ranging from highly polar molecules like short chain carboxylic acids, amino acids and sugars to highly apolar lipids, such as sterols. Their structural diversity includes linear, cyclic, and multiring configurations with various bond types.^119^ A notable example are lipids, often considered a uniform group but exhibiting high molecular diversity, posing analytical challenges for LC–MS due to isomerism.^120^ Due to this complexity, no single analytical method can cover the entire metabolome/lipidome.

Consequently, liquid chromatography strategies in metabolomics have evolved from predominantly single-mode LC separations toward the combination of complementary chromatographic techniques. Due to its robustness and reproducibility, RPLC remains the most-used technique in LC–MS applications.^27,106^ However, its limitation on capturing increasingly diverse and polar compound classes prompted the broader adoption of HILIC, IEC, and other specialized separation modes. More recently, multi-LC strategies combining orthogonal separation mechanisms,^44–46^ such as RPLC and HILIC, have been widely employed. The resulting large-scale targeted LC–MS methods identify and quantify hundreds of metabolites for routine analysis of livestock samples, thereby enhancing metabolite coverage. This trend marks a transition toward emphasizing coverage-driven and application-specific method optimization in modern LC–MS metabolomics. Within our survey, only 4 out of 36 methodology clusters combined different LC techniques to enhance the metabolic coverage (Table S2, clusters 1,3,4,5). This outcome was expected, as most targeted LC–MS studies focused on a limited set of metabolites within a few compound classes, for which a single separation technique is typically adequate.

With the goal to maximize the metabolic coverage, orthogonal chromatographic techniques are recommended. However, only two clusters combined RPLC-MS and HILIC-MS,^28,81^ which significantly enhanced the metabolic coverage, enabling the quantitative determination of 235 metabolites spanning 19 compound classes in animal plasma.^28,76^ Similarly, AEX and RPLC-MS were combined only by one research group (Table S2, cluster 3). In this approach, AEX-HRMS was employed to quantify polar phosphorylated metabolites and carboxylic acids. Meanwhile, three RPLC methods using a short C18 column covered amino acids and biogenic amines in SRM mode (positive ionization mode after derivatization), bile acids and long chain fatty acids (negative ionization mode) and lipids (positive ionization mode). Additionally, three RPLC methods were developed for semiquantification of lipids from various classes without reference standards, in contrast to the direct flow injection commonly used in commercial kits. The lipid classes included acyl carnitines, phosphocholines, ceramides, cholesterol esters, sphingomyelins, diglycerides, and triglycerides. Lipids from each class were semiquantified based on one or 2 M calibration functions established from reference standards of the same compound class. This approach significantly increased the metabolic coverage, encompassing 349 quantified metabolites, and 523 semiquantified lipids.

To further improve metabolic coverage in targeted LC–MS workflows, future livestock studies should employ complementary separation techniques. A major challenge, however, is that combining LC–MS methods often entails a trade-off between coverage and analysis time. Achieving comprehensive coverage of the metabolome or lipidome typically requires multiple methods, which inevitably increases the total analysis time. Some clusters describing the use of several LC–MS methods require different sample preparation for each method, further complicating the workflow. This is exemplified by the article of Mann et al.,^81^ who utilized three LC–MS methods, each with tailored sample preparation protocols, to quantify serum bile acids, coenzyme Q10, and plasma lipid mediators in equine samples. Combing multi-LC methods allows independent optimization of each separation technique. The shortcomings are multiple sample preparation methods, high sample consumption, lengthy analysis times, and laborious efforts including column/solvents exchange, which prevent high throughput or automation.^121^ The setup of online techniques combining different columns within one single injection is technically more difficult—but feasible, which is detailed elsewhere.^122,123^ To further increase throughput without impairing metabolic coverage, fast polarity switching and scheduled/timed/dynamic SRM (sSRM) can be coupled to measure both polarities within one injection and maintain sufficient dwell time. The application of short LC–MS methods (<10 min) is another alternative solution to improve the throughput. This is achievable by using shorter columns, higher flow rates, enhanced temperature, and adjustment of LC gradient and MS settings, enabling analysis of hundreds of samples per day.^124^ However, the increasing application of multi-LC techniques in large-scale targeted methods led to a significant increase in both data volume and complexity. Consequently, integrating the vast and method-specific data sets becomes essential. This involves aligning chromatographic retention times, normalizing quantitative outputs, and, when ISs are employed, their consistent application across all methods.

### 3.4 Employed MS Instruments and Modes

In targeted metabolomics, the most widely used instruments are low-resolution MS (LRMS), like QqQs and quadrupole-ion traps (QTraps). These instruments provide advantages over HRMS in terms of cost-effectiveness, sensitivity, and analytical robustness.^125^ In our survey, LR QqQ (41%) and QTraps (41%) were the most widely employed analysers, followed by Orbitraps (6%) and QTOFs (10%). Meanwhile, 2% of the total methods did not specify the used MS instrumentation. While QTraps offer ion trap capabilities, they were solely operated in conventional QqQ modes. SRM was the most commonly used strategy on LRMS platforms for quantifying hundreds of metabolites. However, as an example, Artegoitia et al.^78^ quantified choline-containing lipid classes in multiple bovine tissues using an adapted HILIC LC–MS/MS method that incorporated multiple scan modes, including the collision-induced dissociation-based precursor ion scan and neutral loss scan in addition to SRM as described in.^101^

HRMS is traditionally favored for untargeted metabolomics due to its extensive metabolic coverage capability and high mass accuracy. With continuous technological refinements in HRMS platforms, including faster scan speeds, enhanced sensitivity, and improved data acquisition workflows, HRMS platforms are finding broader application in targeted metabolomics as well.^43,126,127^ Parallel reaction monitoring (PRM) is one of the techniques used for targeted quantification on HRMS platforms.^128^ Unlike SRM, which monitors only one ion transition of precursor ion to product ion, PRM analyses all product ions formed from the precursor ion.^18^ Consequently, PRM offers higher specificity and provides more detailed information in MS/MS spectra. For instance, Stella et al. quantified four potential biomarkers associated with growth promoter feeding in bovine liver by a PRM-based method using a high-resolution Q-Orbitrap instrument.^129^ Notably, HRMS was also employed in a hybrid approach of merging untargeted and targeted metabolomics approaches to discover and validate potential biomarkers between control and treatment group (chapter 4.3). Additional applications include an AEX-Orbitrap method for quantifying anionic compounds like carboxylic acids in livestock biospecimens^89^ and a data-independent acquisition (DIA) approach on a QTOF for lipid quantification in bovine heart extract, which demonstrated enhanced speed and specificity for complex mixtures.^130^

### 3.5 Method Development, Quantification, and Validation

Targeted LC–MS studies apply different quantification strategies, depending on analytical objectives and standard availability. Relative quantification, based on comparison of signals (usually peak areas normalized to ISs or drift corrected using quality control samples) from different samples, is a common strategy in large-scale metabolite panels or when authentic standards are unavailable.^81,103,131^ On the contrary, absolute quantification enables much more robust interstudy comparability, cross-platform validation, and establishment of physiologically meaningful reference ranges that promote biomarker discovery. The most common form is to use external calibration curves generated from authentic standards. Alternatively, standard addition can be used to correct for losses during sample preparation and/or mass spectrometric matrix effects, depending on the time point when standards are added. However, this requires multiple workup or injections of one given sample. Finally, internal standards (ISs) can be added to samples, requiring just a single injection for matrix effect compensation. Isotope-labeled ISs are chemically identical to the target analytes, but can be differentiated due to their increase in mass. They are generally favored for their ability to compensate for analyte losses during sample preparation, and to compensate for matrix effects during ionization. When compound-specific standards are unavailable, semiquantification using surrogate calibration (e.g., a member of the same compound class) provides practical means to estimate concentrations of structurally similar metabolites.

A major challenge in method development is the necessity of using reference standards, which are essential to determine retention times, compound specific MS parameters and to perform quantification and method validation. Especially in the case of lipids which comprise diverse combinations of backbones and fatty acids, the majority of metabolites are not commercially available as reference standards. The need to acquire reference standards and to prepare suitable reference standard mixes can partially explain the low percentage of large-scale methods currently available for targeted animal metabolomics (26%). Without authentic standards, identification confidence is reduced. As reference standards are consumed in every measurement sequence, the quantification of a large number of metabolites is resource-intensive and costly.

A time-consuming challenge in method development is the need to optimize SRM transitions for analyte acquisition. During SRM transition optimization, different precursor ions (e.g., [M + H]^+^, [M + Na]^+^, [M + NH_4_]^+^, or [M–H]^−^, [M + CH_3_COO]^−^) exhibit different intensities. After selecting the preferred polarity, usually [M + H]^+^ or [M – H]^−^ ions are used as precursors for fragmentation. Therefore, optimization of the source declustering potential and of the collision energy in the fragmentation cell is important to gain a high intensity of the target analytes.

Our analysis showed that of the total methods covered in this review, only 26% acquired multiple SRM transitions per analyte, 43% relied on only one transition per analyte and 31% of the reviewed methods did not report the SRM transitions used. The trend is even more pronounced in practice, as commercial kits usually also utilize only one transition per analyte. It is conceivable that using a single SRM transition per analyte simplifies method setup, enables faster data evaluation, and allows more analytes to be included in a single method without compromising cycle times. However, this approach of using only one transition increases the danger of total misidentification or improper quantification due to isomeric or isobaric compounds producing the same fragment. For example, the isomeric compounds α-aminobutyric acid (AABA), β-aminobutyric acid (BABA), and γ-aminobutyric acid (GABA) have similar fragmentation patterns as the isobaric metabolites choline and *N*-methyl-alanine, and elute at comparable retention times in HILIC, potentially leading to misidentification if only a single transition is monitored per analyte.^28,76^ Finding and including additional unique transitions is necessary to resolve this complexity. The ion ratio, defined as relative abundance of qualifier ion to quantifier ion, serves as an additional identification parameter that, when combined with RT, enhances the reliability of compound identification. Using multiple SRM transitions, however, decreases dwell times, which is especially pronounced in large scale methods and results in higher signal variance. Strategies such as sSRM to maintain sufficient dwell time by monitoring each transition within designated time windows or splitting analytes across multiple methods can be utilized to mitigate this limitation.

Surprisingly, we also observed a gap in the documentation of LC–MS methods. This was demonstrated by the finding that one-third of the reviewed methods did not report the SRM transitions used, while 2% did not indicate the used MS instrumentation and 17% lacked details on the LC method (see Figure 2). This documentation gap significantly undermines method reproducibility and limits the methods’ applicability within the broader livestock and analytical science communities.

Robust method validation is essential to ensure reliable and accurate quantification. This includes the assessment of apparent recoveries, repeatability, trueness, limits of detection (LODs), lower and upper limits of quantification (LLOQs and ULOQs), linearity, and stability. Close inspection of the LC–MS methods used for targeted animal metabolomics revealed that many of these methods were either only partially validated or not validated at all (Figure 3). Overall, 35 out of 51 targeted methods were employed for quantitative analysis without undergoing validation, including one method that only reported LODs. We defined full validation as including, at a minimum, the assessment of key validation parameters such as LODs, LLOQs, ULOQs, apparent recoveries, and repeatability. Based on this criterion, only six of the 16 validated methods were fully validated, while the remaining 10 were considered partially validated. Notably, eight methods were originally validated in a different matrix and later adapted to the current studies.

**Figure 3:**
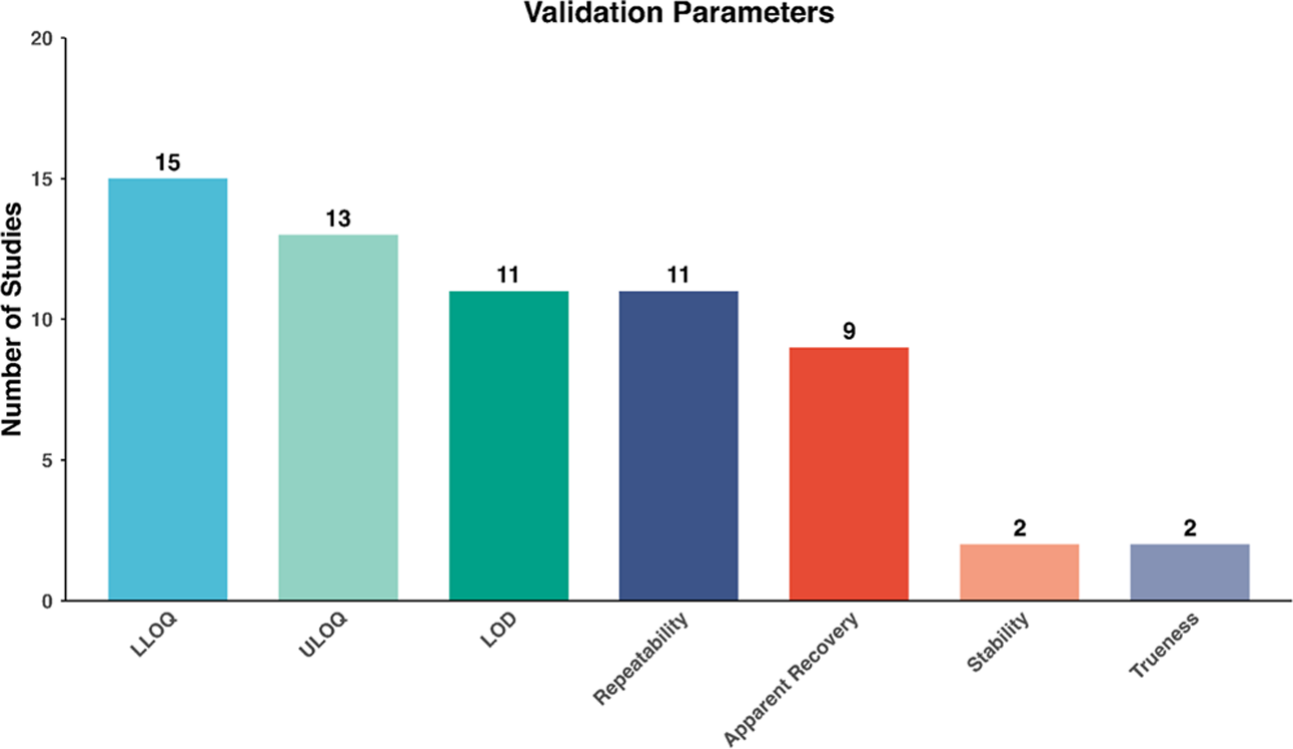
Number of validation parameters covered by targeted LC–MS methods (51 targeted LC–MS methods in total).

Several factors contribute to the low proportion of partially and fully validated methods. Apart from the requirement of obtaining reference standards, another obstacle is the lack of analyte-free matrices. Biological matrices contain endogenous metabolites with concentrations spanning several orders of magnitude, which complicates validation procedures, especially if spiking experiments are required as for the determination of apparent recoveries. To address this, surrogate matrices can be used as synthetic alternatives of the biological matrix, including pure solvent and artificial matrices. Artificial matrices should incorporate the key components of the real sample, including those that contribute to ion suppression or enhancement. Although this approach has been used in urine analysis,^132,133^ it remains challenging to imitate the other complex matrices, such as plasma or tissue homogenates.^134^ Alternatively, authentic matrices mirroring the composition of biological samples are also used for method validation, with endogenous metabolite levels corrected via peak area subtraction,^135,136^ biological matrix dilution,^135,137^ or depletion of endogenous compounds.^135,138^ However, such approaches have several limitations, as discussed in detail elsewhere.^139^ The major drawback is their incomplete mimicry of the actual matrix, along with the high costs and limited commercial availability of suitable materials.

LC–MS techniques are prone to mass spectrometric matrix effects, which arise when coeluting (in SRM mode undetected) endogenous metabolites of the sample matrix enhance or suppress the analyte ionization compared to that in pure solvent.^26^ They can seriously impact accurate quantification, when not corrected for. Interferences can also arise from exogenous contaminants introduced during sample preparation (e.g., plasticizers or Li-heparin from sample containers).^140,141^ Matrix effects are compound-specific, and the chemical property of metabolites and coeluting matrix affects their severity.^142^ Incorporating ISs is a widely recommended practice to correct for the variations introduced during sample preparation and LC–MS analysis.^143^ This is typically accomplished by adding a predetermined amount of IS to each sample before further processing. A constant IS level across samples allows for evaluating analytical variability or matrix effects on a sample-by-sample basis.^144^ Overall, in 63% of the total methods, ISs were added at some point during sample preparation. The optimal stage at which to introduce ISs depends on the intended function of the IS. ISs can be added at the start of sample workup to cover all steps from sample preparation to LC–MS analysis. More often, ISs are added prior to LC–MS analysis to compensate for ion suppression/enhancement, varying adduct formation, or instrument drift, ultimately promoting reliable absolute quantification.^71^ Here, 69% of IS-utilizing methods added the ISs before sample preparation, 9% during sample preparation (e.g., before vacuum drying,^145^ centrifugation and filtration^84^), 19% prior to LC–MS analysis and 3% did not specifying the time point of IS addition. Of the 32 methods that used IS, 26 employed isotopically labeled ISs, while six used native, structurally analogous metabolites. This agrees with previous findings that isotopically labeled ISs, such as deuterated or ^13^C-labeled ISs, are typically favored in LC–MS/MS methods compared to surrogate ISs, which are nonendogenous metabolites with similar chemical properties.^144^ Due to their very similar physicochemical properties, isotopically labeled ISs closely mimic the behavior of the target metabolites during extraction, chromatographic separation, and MS analysis but are distinguished by their specific *m*/*z*.^146^ However, it is challenging to include a compatible stable isotopically labeled IS for each metabolite because of limited commercial availability and partly high costs. This is especially true in large-scale metabolomics assays targeting hundreds of metabolites from various chemical classes. As a compromise, many methods perform absolute quantification using a single IS per metabolite class, leading to reduced accuracy compared to methods where each analyte has its dedicated isotopically labeled IS, unless correction factors are determined in the course of proper validation.^147^ As mass spectrometric matrix effects are caused by coeluting matrix compounds affecting the analytes’ ionization, ISs supposed to correct for matrix effects should be used only for metabolites eluting at a very similar retention time (RT). ISs can be purchased as single compounds or as predefined mixes, like deuterated amino acid mixes. The drawback of IS mixes is the greater danger of introducing impurities. For instance, ISs and impurities from IS mixes can interfere with SRM transitions of naturally occurring compounds, leading to unreliable quantification, as observed with glycine and histidine when a deuterated IS mix was used.^92^

Apart from proper use of ISs, the inclusion of apparent recovery data can also correct for matrix effects in targeted LC–MS platforms. Determination of apparent recoveries usually involves adding defined amounts of standard compounds to the sample before sample workup.^148^ In the absence of a blank matrix, standard addition is used. It typically involves two steps: an initial estimation of the unknown analyte concentration, and the actual standard addition where defined amounts of reference standards are spiked into the sample before workup and slopes of standard addition curves and pure solvent calibration curves are compared.^149^ This process is labor-intensive and requires large quantities of standards and sample material. Additional challenges, particularly in cases of high natural concentrations, include issues with standard solubility, and the linearity of the calibration curves in sample matrix.^28^ In total, 16% of the total methods reported apparent recovery data. This proportion remains relatively low, considering that correcting for the apparent recoveries is critical for ensuring the reliability and accuracy of bioanalytical methods.^150^

Trueness is another barely validated parameter. Trueness is the closeness of the measured value to the “true” value of a CRM. The only two methods that partly assessed trueness used a human plasma standard reference material (NIST, #1950) as a surrogate of porcine plasma, considering the physicochemical similarity between the two species.^28^ The absence of a CRM with concentrations for a large metabolite panel in animal plasma is a major cause of such a low trueness validation rate. In view of this, bioanalytical methods for endogenous metabolite quantification should be cross-validated against a well-established reference method to ensure accuracy and reliability.^139^ Based on our survey, two methods compared their measured results with those obtained from a reference method. For instance, one AEX-HRMS method was used to cross-validate two RPLC-MS/MS methods using different derivatization reagents for quantifying carboxylic acids in animal samples.^89,151^ In another study targeting ubiquinol-10 and ubiquinone-10 in human plasma, measured values were compared to those obtained through chemical oxidation with 1,4-benzoquinone, demonstrating excellent agreement.^152^ As a result, this method was subsequently adopted for equine serum samples.^81^ In a later study, one derivatization-based RPLC-MS/MS method and one protein precipitation plus dilution-based HPLC-MS/MS method were compared for quantification of amino acids, amino acid related compounds and biogenic amines.^92^ This comparison showed that derivatization enhanced isomeric separation and minimized carryover but was associated with lower repeatability, in part multiple derivatization products, and impaired determination of nonderivatized compounds. In contrast, the dilute-and-shoot approach proved more reliable for nonderivatized analytes. Overall, application of both methods to plasma from multiple species yielded largely comparable metabolite concentrations. Finally, commercial kits can be also employed to cross-validate the results from other platforms, as discussed above.

Special care should be taken when transferring methods designed for a specific matrix to other matrices. Due to different compositions, also matrix effects will be different. When no compensation strategies, such as ISs, are used, the applied method can only be regarded semiquantitative, unless it has been validated for the new matrix as well.

Ultimately, another challenge related to LC–MS method validation includes the requirement for periodic revalidation to maintain the continued reliability and quality of the clinical data.^153^ A comprehensive validation is often extremely time-, resource-, and labor-intensive, especially when targeting hundreds of metabolites across diverse compound classes and multiple sample types. Therefore, periodic revalidation focusing on the most critical parameters—including LODs, LLOQs, ULOQs, matrix effects, and repeatability—is a pragmatic compromise for large-scale targeted metabolomics method, which maintains both analytical reliability and practical feasibility.

### 3.6 Comparison of Applied Methods

Developing novel LC–MS methods is a time-consuming process, especially when targeting hundreds of chemically diverse metabolites. To help with method development, transferring LC and MS parameter data sets from the existing literature is a practical approach. SRM transitions and LC methods are often transferable even across different LC–MS/MS platforms. To some extent, this might also apply to validation results, provided the same ion source is used and the validated matrix is inherently similar to the new target matrix. However, careful verification and, if necessary, reoptimization of SRM transitions and chromatographic conditions, and revalidation remain essential to account for potential matrix effects, instrument-specific differences, and retention time shifts.

The 51 methods selected in this review were classified into “small-scale targeted methods” (number of metabolites <100) and “large-scale targeted methods”. Overall, 14 of the included methods (27%) targeted less than 20 metabolites, and 23 methods (45%) covered between 21 and 99 metabolites. This high percentage is mainly because the majority of targeted studies focused on quantifying only a few metabolite classes associated with specific biological questions. Among 37 small-scale targeted LC–MS methods, the majority was not validated at all, 9 methods were partially validated and only four methods were fully validated. Three out of the 13 validated small-scale targeted methods used two or more SRM transitions per analyte. Notably, only one method was fully validated and used two transitions for analyte acquisition.^154^ Meanwhile, 13 LC–MS methods (26%) met the criteria for large-scale targeted metabolomics, while one method did not specify the targeted metabolite number. However, the validation status of large-scale methods was poor, as only two of them were fully validated.^28^ In addition, five methods lacking clear LC configuration, or complete SRM transition data were excluded due to limited applicability for the livestock community. The remaining nine large scale targeted methods are categorized into 3 methodological clusters, summarized in Table 1. Provided that complete method descriptions are available, large-scale methods offer greater utility than small-scale methods due to the availability of extensive data sets of LC and MS parameters for targeted livestock methodology development.

**Table 1:** Comparison of Large-Scale Targeted LC–MS Methodology Clusters

reference	no. of metabolites	metabolite classes	total run time (min)	LC–MS methods	no. of transitions per analyte	validation	additional information
^28,76^	235	17 compound classes	RP: 23.0; HILIC: 16.5; total: 39.5	RP-MS/MS + HILIC-MS/MS	2	full validation	protein precipitation plus dilution-based sample preparation
^156^	258	diverse polar metabolites	15	HILIC-MS/MS	Partially 2, majority 1	no validation	workflow guidance
^110^	872	20 compound classes	pos: 7.5; neg: 9.0; lipids: 12.0; lipids w/o stds: 3 × 10.0; AEX: 28.0; total: 86.5	6 RP-MS/MS, 1 AEX-HRMS	2 for 249 compounds with standards; 1 for 523 lipids w/o stds; HRMS in full scan mode for AEX	no validation except for partial validation of the AEX-HRMS method	derivatization of amino acids and biogenic amines

Based on a prior comparison of seven targeted LC–MS methods,^44^ Xu et al. developed a combination of RP- and HILIC-MS/MS methods for identifying and quantifying 235 metabolites spanning a large number of compound classes.^28,76^ In contrast to the other three large-scale methodological clusters, this cluster’s methods were fully validated in porcine plasma, demonstrating minimal carryover, excellent linearity and repeatability, and high trueness. Although not including ISs, apparent recovery data guarantee reliable quantification by correcting matrix effects and potential analyte losses during sample preparation and cleanup.^150^ Additionally, it was the only cluster acquiring every analyte with two SRM transitions. The LC run time was extended to wash the column with strong eluents, reducing carryover in daily routine analysis. Two articles^102,155^ adopted a high-pH HILIC-MS/MS method established by Yuan et al.,^156^ in order to quantify 258 polar metabolites. This method provides a step-by-step workflow from dedicated sample preparation for various matrices to LC–MS condition setup, measurement, data evaluation, and application in a case study. It is a rapid, single 15 min HILIC method using fast polarity switching, and ideal for studies investigating the polar metabolome. Its major drawbacks are the acquisition of most compounds using only one SRM transition and the absence of method validation.

One research group used a cluster of seven different LC methods (six RPLC-MS/MS methods and one AEX-HRMS method) to achieve the highest metabolic coverage of 872 chemically diverse metabolites in several livestock studies, with 349 metabolites absolutely quantified and 523 lipids semiquantified.^32,111,112,115,116^ The comprehensive set of SRM transitions and LC setups is transferable to other LC systems, although further validation in real matrices is warranted. In a later study, the PITC derivatization based RPLC-MS/MS method for determination of amino acids, amino acid related compounds and biogenic amines was slightly modified and thoroughly validated in porcine plasma.^92^

To summarize, it is an efficient strategy to adapt existing large-scale methods to specific analytical requirements or sample types. Although minor adaptations and subsequent validation may be necessary, the time-consuming steps of SRM parameter and LC separation optimization can be largely circumvented.

## 4 Trends in Targeted LC–MS Based Metabolomics for Livestock Research

### 4.1 Research Questions

We classified the selected articles according to their research aims, which are detailed in Table S1. Animal health achieved the highest percentage of 35% of the total articles (23 out of 66 studies), making it the most prominent category. LC–MS metabolomics methods have proven to be a powerful tool for disease diagnosis and prognosis, promoting livestock health, and disease resistance. Disease control is pivotal in efficient livestock production and economical livestock management. A purulent strategy of disease control is to prevent its onset through early detection. Timely prediction of disease enables the implementation of cost-effective preventive measures, which are more economical than treatment after diagnosis and enhance animal welfare. Additionally, effective surveillance and early intervention are key to preventing disease outbreaks, boosting production performance, and safeguarding animal health in the livestock sectors. A total of 4 articles were related to disease diagnosis, such as detection of Huntington’s disease in transgenic sheep^30^ and leukemia virus in cows.^57^ Two articles were related to the prediction of subclinical mastitis^29^ and ketosis^58^ in dairy cows. Targeted metabolomics revealed that ketosis is associated with coordinated alterations in amino acids, phospholipids, and biogenic amines, enabling identification of predictive and diagnostic biomarkers distinguishing ketotic from control cows. Similarly, subclinical mastitis showed disruptions in amino acid and lipid metabolism, with lysine, leucine, isoleucine, kynurenine, and specific phospholipids identified as key biomarkers. These metabolic changes were detectable weeks before clinical onset, underscoring the value of targeted LC–MS for early disease prediction. Considering the importance of disease prediction, future efforts in this area should be expanded. Other examples of targeted LC–MS methods used in the area of animal health included identification of metabolic biomarkers in response to physiological disturbance or stressors, for instance exposure to heat or excessive lipolysis during lactation in bovine, and introduction to a high-intensity training in horses.^60,82,157^ Additionally, diverse livestock species were challenged with toxins,^158,159^ or drugs.^90,129^ Metabolomic and lipidomic analyses identified key biomarkers distinguishing heat-stressed dairy cows, reflecting altered pathways in carbohydrate, amino acid, lipid, and gut microbiome metabolism and highlighting the potential of targeted LC–MS for heat stress diagnosis.^82^ Additionally, one venom toxin exposure study in livestock model revealed significant perturbations in several metabolite pathways and amino acid metabolism, with metabolites such as glutamine serving as potential, albeit nonspecific, diagnostic biomarkers.^159^ These examples highlight how pathological and physiological challenges induce metabolic changes in livestock, which can be captured using targeted metabolomics approaches.

*Animal nutrition* examines the nutritional composition of feed and dietary demands of various livestock species. Livestock nutritional metabolomics studies the effects of various dietary factors on metabolism, including specific diets, feeds, nutrients, micro-organisms, or bioactive compounds. In response to nutrients, metabolites are influenced directly or indirectly in the tissue and body fluids, and can serve as potential biomarkers.^160^ Seventeen articles in our survey (26%) focused on animal nutrition, ranking as the second largest research area. Overall, a large proportion of articles investigated the effect of certain feeding practices on the livestock metabolome, such as creep feeding and weaning or fructose-rich diet in swine,^116,161^ or diet with surplus energy and protein or high-grain in bovine.^79,111^ Some of these diets are linked to the occurrence of abnormal status of livestock species, including diet-induced inflammation, rumen acidosis, and obesity. To counteract the challenge of these specific diets, feed additives have been widely explored as nutritional interventions to improve digestion and gut health in livestock. For example, a clay mineral-based feed additive was supplied to cows fed with high-starch diet, leading to increased primary and secondary bile acids, which enhanced the liver function.^31^ Similarly, phytogenic feed additives were added in the diet of cattle fed with high grain diet, resulting in reduced levels of potentially harmful metabolites (e.g., spermine and spermidine) generated due to ruminal dysbiosis.^32^

*Animal products*, as the third largest research area, were the topic of 6 (9%) of all included articles. The research area includes studies focusing on the final product (e.g., meat, milk, cheese) and their quality for human consumption, in contrast to animal production (4% of the included articles) that investigates metabolic processes within the living animal influencing production traits. The relatively high percentage of animal product studies aligns with the growing area “foodomics” that focuses on food and nutrition research using -omics technology, consequently benefiting human health.^162^ One major application in animal products is quality characterization of various animal-derived products. For example, a targeted LC-MS-based metabolomics analysis was conducted to profile amino acids and their derivatives in yak colostrum and mature milk, revealing a higher nutritional value in yak colostrum due to its abundance of functionally active amino acids.^99^ Another article highlighted the difference of quantified pyruvate and TCA cycle related metabolites in bovine longissimus lumborum and *psoas major* muscles, indicating distinct *post mortem* energy metabolic patterns that ultimately affect meat quality throughout the supply chain.^37^ Other applications included metabolic profiling of bioactive compounds (e.g., oligosaccharides) in animal products.^100^

Primarily characterizing the metabolome of specific organs, tissues or body fluids, animal physiology accounted for 9% of the selected articles, whereas animal reproduction accounted for 8%. Notably, all four articles (6%) associated with *human health* were conducted using swine as a model to investigate human health due to the similarity of the two species regarding physiology, anatomy, immunology, and genome.^163^ For example, infant porcine models of cardiopulmonary bypass and deep hypothermic circulatory arrest revealed disruptions in energy mechanism and altered amino acid, carbohydrate, and redox metabolism pathways associated with acute lung injury observed in humans.^155^ Similarly, juvenile swine models of metabolic syndrome showed altered glycolysis-related pathways and impaired myocardial energy metabolism, including disruption of the glucose–G6P–pyruvate axis and reduced ATP availability, mirroring key metabolic alterations observed in human metabolic syndrome and cardiovascular disease.^102^ These findings demonstrate that swine metabolomics extends beyond species and provides translational insight into disease-associated metabolic perturbations for human clinical diagnosis and treatment development. *Methodology* represented another small category, comprising only 6% of the articles, with only four papers fitting in this group. The low percentage may be partly attributed to the common utilization of commercially available targeted kits in livestock science. Interestingly, studies dedicated to the development and validation of targeted LC–MS methods in livestock metabolomics are still scarce, contrasting with the booming of large-scale targeted LC–MS methods in human or rodent studies.^47–52^ Besides the two articles previously discussed in this review,^28,89^ a novel data-independent acquisition method using a rapid scanning quadrupole coupled to a TOF mass analyzer (SONAR) was developed, offering high-quality quantitative data for lipid analysis in bovine heart extract.^130^ Narduzzi et al. compared three different RP columns and selected RP-F5 due to its better performance in separating isomers of bile acids.^97^ This method was further applied to pig serum samples for quantifying bile acids, determining the link of reduction in bile acids after exposure to polychlorinated biphenyls. Greater research efforts should be directed toward developing targeted LC–MS methodologies specifically tailored for livestock metabolomics.

### 4.2 Livestock Species and Sample Types

Among the 66 articles collected through the literature screening, the majority focused on a single livestock species, while three articles investigated multiple livestock species. Due to the economic importance and high production scale of bovine and swine, these species accounted for 49% and 34%, respectively, of all reviewed articles. Additionally, a wide range of targeted studies were performed in bovine and swine across various fields, for instance animal health, dietary effect of feed additives, and characterization of animal products. Oppositely, only 7% and 8% of total articles investigated horse and sheep using targeted LC–MS methods, respectively, with goat being the least studied (1%). This trend of bovine and swine as the most extensively studied livestock species is illustrated in Figure 4. Apart from a disparity in research focus across livestock species, an uneven distribution of studied sample matrices was also observed. Among the 66 articles, 11 articles focused on more than one sample type. Body fluids (e.g., plasma, serum, saliva) accounted for 63% of the analyzed matrices, followed by organs or tissues (e.g., heart, liver, and meat) at 29%, excreta (urine and feces) at 5%, and digesta (content of cecum, duodenum, and ileum) at 3%.

**Figure 4:**
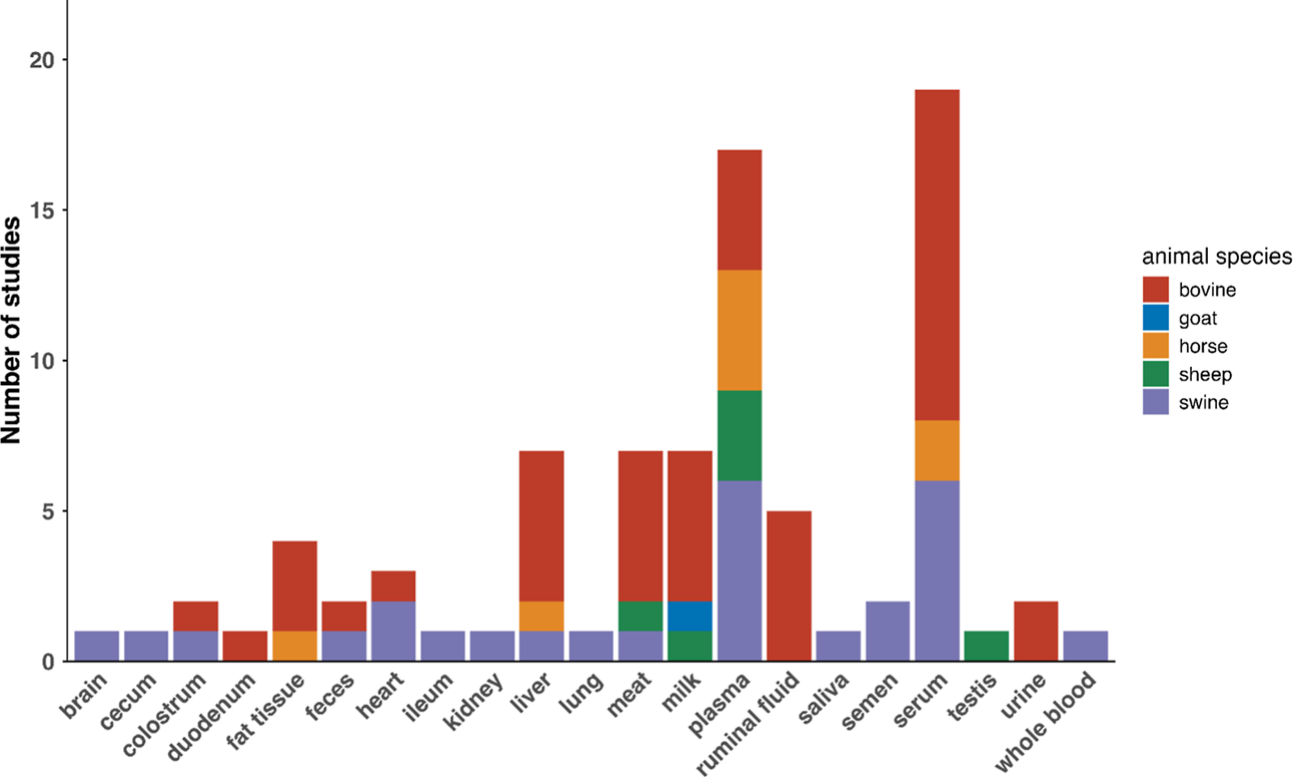
Overview of different sample types and livestock species analyzed by livestock metabolomics studies. Ileum, cecum, and duodenum refer to the digesta content of these organs.

Serum and plasma accounted for the largest proportions of total articles, representing 22% and 20%, respectively. This is in line with human plasma and serum as the most widely used body fluids in metabolomics investigations.^164^ Milk and meat, as essential animal products, were also frequently studied, both achieving 8%, respectively. In contrast to cow milk, goat and sheep milk were understudied given their global economic importance (see Figure 4). Human milk oligosaccharides (HMOs) have been shown to contribute to the development of infant’s immune system during breastfeeding^165^ and may also enhance cognitive functions beyond infancy.^166^ Therefore, one article compared the oligosaccharide profiles of goat, bovine, sheep, and human milk in terms of diversity and content, concluding that sheep and goat whey could serve as valuable sources of oligosaccharides if exclusive breastfeeding is not feasible.^100^ Semen and testis were associated with animal reproduction research, but they were only researched once in swine and sheep, respectively. Feces, colostrum and three digesta were among the least studied sample types in targeted metabolomics. This is surprising as feces provides valuable insights into diet-microbiota-host interaction as it contains unabsorbed metabolites.^167^ The only study on fecal samples used targeted LC–MS/MS to determine that arachidonic acid (ARA)-derived oxylipins, particularly 12-HETE, were more abundant in the feces of suckling piglets at day 3 than at day 21 postnatally, indicating a role of ARA metabolites in early developmental changes.^98^ Likewise, saliva metabolomics was barely explored compared to other fluids, even though saliva is rich in bioactive compounds such as nucleic acids, amino acids, and sugars, and easily collected, with its metabolite shifts offering potential value for disease diagnosis and prognosis in porcine speies.^168^ Except for liver (8%), tissues were generally underrepresented, with brain, kidney, and testis among the least frequently studied.

Body fluids can often be collected minimally invasive and are considered as a more convenient sample type for analysis compared to tissues. This can also be attributed to a less complex sample preparation than for tissues. However, body fluids are nonorgan specific and provide insight into many biochemical processes over diverse tissues in the body.^169^ Oppositely, tissue metabolomic studies have the advantage of providing deeper insights into organ-specific abnormal metabolic processes occurring directly at the site of disease development, aiding in biomarker discovery for diagnosis and prediction.^170^ Given their potential to reveal localized metabolic alterations and improve biomarker discovery, tissues remain an underutilized resource in metabolomics research and merit greater attention in future studies. Overall, an uneven research focus across livestock species and sample types continues to limit progress; addressing this gap is crucial to achieving a more comprehensive characterization of the livestock metabolome.

### 4.3 Combination of Untargeted and Targeted Approaches

Hybrid approaches of combining targeted and untargeted metabolomics, either within a single platform or across multiple instruments, are emerging in livestock metabolomics. These approaches allow for accurate quantification of predefined metabolites, while also discovering new and possibly unexpected compounds, contributing to a comprehensive and informative metabolic profile. Therefore, combining targeted and untargeted metabolomics retains the key advantages of both approaches, while mitigating some of the limitations.^18^ In addition, this approach supports both hypothesis-driven and discovery-driven research, enabling a more holistic picture of the biological system within a single study. Out of the 66 articles reviewed, 14 employed such a hybrid approach, and 11 of these utilized LRMS and HRMS in combination. Typically, one HRMS instrument is applied for untargeted metabolomics, while an LRMS instrument is dedicated to targeted metabolomics. Overall, this dual-instrument setup provides higher metabolic coverage than single approaches.^171^ In contrast, three articles used a single HRMS instrument for hybrid measurement approaches. Stella et al. utilized a Q-Orbitrap to perform untargeted metabolomics with the aim to discover potential biomarkers related to growth promoter administration in bovine liver tissue. Key metabolites were quantified using a full-scan method for screening and PRM for quantification on the same instrument.^129^ Single-instrument hybrid setups offer several advantages over dual-instrument hybrid setups. The advantages include lower acquisition and maintenance costs, reduced sample requirements (crucial with limited sample volume), and lower efforts needed for instrument parameter reoptimization between platforms. Combining targeted and untargeted metabolomics can be considered as an all-in-one workflow. It facilitates the discovery and validation of biomarkers between treatment and control conditions, pathway mapping of key discriminant metabolites, and obtaining deeper insights into complex biological processes.

All 14 articles employed untargeted metabolomics to discover new or unexpected metabolites, and key metabolites were further quantified for validation in targeted analysis. For example, in a study to determine early pregnancy biomarkers in sows, hyodeoxycholic acid and 2′-deoxyguanosine were identified as potential early pregnancy biomarkers through untargeted metabolomics analysis of saliva samples. These two compounds were evaluated by targeted LC–MS/MS methods for quantification and ROC curve analysis, confirming their diagnostic effectiveness.^172^ Yet, combination of targeted and untargeted approaches also has shortcomings.^171^ These include long sample run times, complex instrument optimization, and time-consuming data evaluation. Limited integration with public databases also hampers confident metabolite identification. Improving throughput, database compatibility, and data filtering strategies is essential to overcome these limitations.

### 4.4 Multiomics Research

Another apparent trend is the integration of targeted metabolomics with other -omics techniques in livestock science. Overall, 13 articles utilized multiomics techniques, including genomics, transcriptomics, proteomics, and microbiomics. Notably, 10 articles used a two-layer omics-metabolomics analysis. This approach focused on exploring the direct relationships between two different omics data sets to identify potential biomarker candidates.^6^ Combining multiple omics techniques with metabolomics generates complex and multidimensional data, which poses several challenges to data integration^8^ and requires advanced tools to correlate data sets across omics layers.^173^ This complexity can partly explain the lower prevalence of integrated multiomics-metabolomics studies in our survey. Among the two-layer analysis in livestock research, microbiomics-metabolomics analysis made up the highest percentage (50%), followed by transcriptomics-metabolomics (30%) and proteomics-metabolomics (20%). Of the three articles using multiomics analysis, two integrated transcriptomics, microbiomics, and metabolomics. In microbiomics, 16S rRNA sequencing is the most predominantly employed technique to characterize gut microbial communities and composition. Microbiomics refers to the research of microbial communities with distinct physicochemical traits that function as dynamic ecosystems.^174^ These communities interact closely with their surrounding environments and host organisms, across varying timelines and scales. Integrating microbiomics and metabolomics offers a better understanding of the complex crosstalk between host metabolism and gut microbiome in livestock science. This combination can also guide dietary interventions aimed at optimizing rumen function and nutrient efficiency, thereby enhancing animal health.^175^ Ricci et al. used microbiomics-metabolomics analysis to investigate how increasing proportions of starch in the diet, with or without phytogenic feed additive supplementation, affect microbial adaptation in different gastrointestinal niches of cattle.^32^ The approach characterized microbial composition and diversity in rumen digesta and feces, quantified ruminal metabolites (e.g., various carboxylic acids, sugar phosphates and biogenic amines) associated with microbial activity, and correlated microbial metabolic pathways to carbohydrate metabolism. In a triple-omics (microbiomics, transcriptomics and metabolomics) study, the correlation of host immune response and microbial-metabolites dynamics were evaluated during dietary transition to high-grain feeding in cows.^111^ It revealed coordinated shifts in ruminal inflammation-associated gene expression, microbial composition, and metabolic profiles, highlighting the interaction between host immune response and gut microbiota during dietary transitions. These studies address the pivotal role of multiomics approaches in uncovering complex host-microbe-diet interplays in livestock. Above all, merging metabolomics results with other omics data facilitates the discovery of candidate biomarkers, elucidation of underlying mechanisms, and a more comprehensive insight into the biological system than single-omics platforms. However, also these advantages come with trade-offs. In addition to the high costs of data generation and the need for substantial bioinformatics support, a key challenge in multiomics lies in the integration and interpretation of complex, multimodal data. Researchers have to address issues such as data overload, tool limitations, high dimensionality, too small number of samples analyzed by several different omics techniques, and added complexity from single-cell and spatially resolved omics.^176^ Nonetheless, the benefits of combined omics far outweigh these limitations when addressed with thoughtful experimental design and appropriate bioinformatics support. Amid rapid advancements in omics data generation and the development of increasingly sophisticated analytical tools, we anticipate a growing expansion of integrated omics-metabolomics studies in livestock research in the future.

## 5 Recommendations

Compared to available commercial kits, development or even implementation of a targeted LC–MS method needs more time in the starting phase. However, it is more sustainable and budget-friendly in the long run, particularly when dealing with a large number of samples on a daily routine analysis basis. Moreover, sample preparation, LC–MS parameters and validation can be tailored for specific analytical needs of livestock studies. This might include avoiding laborious derivatization, when using a combination of LC conditions. Depending on the size and chemical diversity of the targeted metabolites, a set of orthogonal separation mechanisms, such as RPLC and HILIC, are recommended to maximize metabolite coverage. Authentic reference standards are analyzed under the selected LC condition(s) to determine the respective retention times, which offers orthogonal information to MS or MS/MS. This not only allows quantification, but also greatly strengthens proper metabolite identification. Likewise, at least two SRM transitions should be selected for each analyte. Fast polarity switching can be applied to accelerate the method, measuring both electrospray polarities in a single run. If possible, ISs should be added to correct for analytical variation, most importantly matrix effects. Method validation is crucial and should at least include recovery, repeatability, LLOQs and ULOQs. Sample randomization is highly recommended to reduce bias from signal drift throughout the run.^177^ Quality control (QC) samples are crucial to verify instrument stability and performance. They are commonly prepared as a pool of small aliquots of the tested samples, representing the average concentration of all analytes. QC samples are measured repeatedly throughout the whole measurement sequence, allowing to monitor instrument drift. An innovative way to handle instrument drift within batches after measurement is to use the open-source application QuantyFey for data evaluation, which offers various methods for drift correction.^178^ Long-term QC samples serve a similar role as QCs, but their main aim is to ensure the monitoring of data quality over time across multiple batches, studies, or laboratories, providing insight into intra- and interlaboratory consistency of LC–MS platforms.^143^ Data evaluation remains a major bottleneck in metabolomics. Modern software such as SciexOS, MultiQuant (both Sciex), MassHunter (Agilent), TraceFinder (Thermo Fisher Scientific) or Skyline (open source^179^), enable peak integration, quantification, statistical evaluation, and reporting, supporting high-throughput workflows in both quantitative and qualitative mass spectrometry analysis. Additionally, specialized data processing software such as MRMPROBS^180^ and MRM-DIFF^181^ are available for the assessment of large-scale MRM data in metabolomics and lipidomics studies. While automated peak integration is essential, time-consuming manual review to ensure reliable quantification, particularly in case of RT shifts, ion ratio mismatch, distorted peak shapes, low-abundance peaks near the LLOQ, and partial coelution of isomeric compounds, is often unavoidable. Despite several limitations, relative quantification is a common practice in targeted metabolomics analysis due to its simplicity and independence from authentic standards and ISs.^43^ Nevertheless, most targeted metabolomics studies rely on calibration-based approaches for absolute quantification. This enables the definition of physiologically relevant reference ranges and allows comparison of data across research sites and study designs.

Beyond quantitative processing, advanced data interpretation strategies are crucial to extract biological insights from targeted metabolomics data sets. Statistical approaches ranging from univariate and multivariate analyses to machine learning models can support biomarker discovery and classification tasks, while pathway analysis facilitates the biological interpretation of targeted metabolite panels. Platforms such as MetaboAnalyst^182^ enable integrated statistical, chemometric, and functional analyses, whereas curated databases (e.g., LMDB^20^ and HMDB^183^) assist in metabolite annotation and interpretation. Lately, pan-repository databases have been compiled, providing MS/MS spectral search across species.^184^ Finally, standardized workflows and FAIR-compliant data sharing frameworks^185^ improve reproducibility, harmonization, and cross-study comparability.

## 6 Outlook and Conclusion

Future research should prioritize the development of methodologies specifically tailored to targeted livestock metabolomics. Greater emphasis should be placed on the establishment of large-scale targeted LC–MS methods capable of quantifying hundreds of mammalian metabolites in a single analysis to reduce run-time, needed sample amount, and reduce instrument contamination. With regards to current limitations of livestock metabolomics application, research is needed to cover other livestock species beyond bovine and swine and explore a greater array of sample types, such as feces, saliva, and various tissues. Emerging trends in targeted livestock metabolomics should be further reinforced, including merging targeted and untargeted metabolomics approaches for biomarker discovery and validation, and integrating other omics techniques with metabolomics to gain a better understanding of biological systems. Table 2 provides a summary of the key analytical parameters that researchers need to consider when developing targeted LC–MS methods. Building on these fundamental considerations, future research should resolve current methodological limitations and establish frameworks tailored to targeted metabolomics in livestock science.

**Table 2:** Summary of Key Features for Targeted LC–MS Method Development

	pros	cons	comment
**Sample Preparation**			
IS	correct for analytical variation or matrix effects	expensive	isotopically labeled ISs favored over surrogate analogues
	can be added at different stages during sample preparation	limited commercial availability	one representative IS for each metabolite class for semiquantification if authentic standards are unavailable
		might introduce interferences	
chemical derivatization	improves LODs	complicates sample preparation	commonly used in combination with RPLC
	expands metabolic coverage	introduces sources of error	
	promotes separation of isomeric compounds	stability issue of certain derivatized metabolites	
DnS technique, protein precipitation and dilution	simple and fast	limited to less complex biological matrices (e.g., serum, plasma, urine)	can be used as orthogonal approach for derivatization-based methods
	low analyte loss	subject to matrix effects	
	broad coverage of metabolite classes	problematic in detecting low-concentration compounds	
	high throughput		
OSE, LLE, SPE	broadly applicable to complex biological matrices	more complex and time-consuming than DnS or protein precipitation and dilution → less suited for high-throughput workflows	selective metabolite coverage compared to broad, nondiscriminant metabolite coverage by DnS or protein precipitation and dilution technique
	enrichment of specific metabolite classes	higher risk of analyte loss	
	enhanced selectivity by removal of matrix interferences		
**Method Development and Validation**			
combination of LC techniques	enhances metabolic coverage	longer analysis time	use of orthogonal LC techniques
	gives rise to large-scale targeted methods	increased sample consumption	
		laborious efforts in sample preparation and measurement	
large-scale targeted LC–MS methods	enable absolute quantification	time-consuming	use of fast polarity switching and sSRM in combination to measure both polarities in one run and maintain enough dwell time per analyte
	target hundreds of metabolites	limited standard availability (e.g., for lipids)	
		decreased dwell time	
full validation	highly reliable quantification	time-consuming	partial validation as alternative (validation of key parameters) for periodic revalidation or when new matrices are measured
	facilitates cross-laboratory comparisons and longitudinal studies	resource-intensive	
	proves method robustness	matrix-specific	
		impractical in large-scale setups	
**Trends**			
multiomics	provides a system-level understanding of biological processes	multidimensional data to integrate and interpret	
	strengthens mechanistic insights and biomarker discovery	demands bioinformatics support and standardized pipelines	
		high cost in generation and analysis	
integration of targeted and untargeted metabolomics	balances discovery of novel biomarkers and quantification of biomarker candidates	complex instrument optimization	
	enables identification of novel biomarkers with subsequent validation	long sample run time	
	supports both hypothesis-driven and exploratory investigation	restricted database availability	

Concluding, targeted LC–MS metabolomics has become a cornerstone in livestock research, enabling quantification of metabolites across diverse biological matrices. Among the 66 recent studies, 25 utilized commercial kits for quantification of several hundreds of metabolites. A total of 51 self-developed LC–MS methods were employed in our survey, showcasing diverse sample preparation protocols, including OSE, protein precipitation and dilution, DnS, LLE, SPE, and chemical derivatization. RPLC was the most commonly used separation technique, followed by HILIC and AEX. Low-resolution mass spectrometers, particularly QqQ instruments, were predominant, while high-resolution platforms were increasingly integrated for hybrid targeted-untargeted approaches. Emerging trends include multi-LC techniques to enhance metabolic coverage, integration of untargeted and targeted workflows for biomarker discovery and validation, and multiomics studies. Research aims span disease diagnosis, dietary effect evaluation, and animal product quality assessment, with bovine and swine being the most studied species and body fluids the most researched matrix. Despite significant advancements, challenges such as limited standard availability, unreliable quantification, lack of validation, incomplete report of LC–MS setups, and uneven research focus across species and sample types highlight the need for further methodological development and standardization in livestock metabolomics.

## Supplementary Material


